# Focus on T cell exhaustion: new advances in traditional Chinese medicine in infection and cancer

**DOI:** 10.1186/s13020-023-00785-x

**Published:** 2023-06-24

**Authors:** Shenghao Li, Liyuan Hao, Junli Zhang, Jiali Deng, Xiaoyu Hu

**Affiliations:** 1 Hospital of Chengdu University of Traditional Chinese Medicine, No. 39 Shi-Er-Qiao Road, Chengdu, 610072 Sichuan Province People’s Republic of China; 2 Chengdu University of Traditional Chinese Medicine, No. 37 Shi-Er-Qiao Road, Chengdu, 610075 Sichuan Province People’s Republic of China

**Keywords:** T cell exhaustion, Traditional Chinese medicine, Infection, Cancer, Immune checkpoint inhibitors

## Abstract

In chronic infections and cancers, T lymphocytes (T cells) are exposed to persistent antigen or inflammatory signals. The condition is often associated with a decline in T-cell function: a state called “exhaustion”. T cell exhaustion is a state of T cell dysfunction characterized by increased expression of a series of inhibitory receptors (IRs), decreased effector function, and decreased cytokine secretion, accompanied by transcriptional and epigenetic changes and metabolic defects. The rise of immunotherapy, particularly the use of immune checkpoint inhibitors (ICIs), has dramatically changed the clinical treatment paradigm for patients. However, its low response rate, single target and high immunotoxicity limit its clinical application. The multiple immunomodulatory potential of traditional Chinese medicine (TCM) provides a new direction for improving the treatment of T cell exhaustion. Here, we review recent advances that have provided a clearer molecular understanding of T cell exhaustion, revealing the characteristics and causes of T cell exhaustion in persistent infections and cancers. In addition, this paper summarizes recent advances in improving T cell exhaustion in infectious diseases and cancer with the aim of providing a comprehensive and valuable source of information on TCM as an experimental study and their role in collaboration with ICIs therapy.

## Introduction

T cell exhaustion is a state of T cell dysfunction, which is mainly characterized by increased expression of a series of inhibitory receptors (IRs), low effector function and reduced cytokine secretion [1, 2]. Moreover, it also accompanied by changes in gene expression related to T cell chemotaxis, adhesion and migration, changes in transcription factor expression profile and metabolic function defects [3]. T cell exhaustion was first observed in mice with chronic viral infection and had subsequently been found in many animal models or patients with chronic viral and parasitic infections and tumors [4].

In the process of acute infection, when antigen is cleared or inflammation subside, effector CD8^+^ T lymphocytes (T cells) further differentiate into functional memory CD8^+^T cells, which produce interferon⁃γ (IFN⁃γ), tumor necrosis factor (TNF), interleukin-2 (IL-2), etc. When secondary infection occurs, effector CD8^+^ T cells can produce strong memory response quickly and efficiently, and carry out immune defense. These memory T cells can also be renewed by IL⁃7 and IL⁃15 driven homeostast to maintain their active state for a long time [5].

In chronic infection, such as hepatitis B virus (HBV), hepatitis C virus (HCV), human immunodeficiency virus (HIV), Corona Virus Disease 2019 (COVID-19) [6] and mycobacterium tuberculosis (MTB) [7, 8], and cancer [9–14], due to chronic antigenic stimulation T cells enter a “exhausted” state. Exhausted T cells lost complete effector function and were highly expressed in a variety of IRs, including programmed cell death protein 1 (PD-1), cytotoxic T-lymphocyte-associated protein 4 (CTLA-4), T cell immunoglobulin domain and mucin domain protein 3 (TIM-3) and lymphocyte activation gene 3 protein (LAG-3) [15, 16]. Exhausted T cells had unique differentiation, phenotype, and function, and stable epigenetic inheritance [17]. In the early stages of infection, virus-specific T cells initially acquire effector function, but gradually deplete and gradually lose their effector function due to persistent viral antigen and inflammatory or infective stimulation [2] (Generally, T cells first lose their ability to proliferate and produce IL-2. In the intermediate stages of cell failure, other T cells properties are lost, including the ability to produce tumor necrosis factor. When the cell is severely exhausted, T cells no longer produce large amounts of IFN-γ or β-chemokines or degranulation. When T cells are completely exhausted, they become exhausted T cells and their effector function is completely lost [18]). However, exhausted T cells are not completely unresponsive but retain some effector functions, allowing the host to control the pathogen without deleterious immunopathology. It turns out that exhausted T cells lead to lethal infections [19].

Reversing T cell exhaustion is critical for inhibiting tumor growth. Exhausted T cells expressed immunosuppressive receptors. Immune checkpoint inhibitors (ICIs) or combination of inhibitor receptors could block these signals, enhancing the effect of immunotherapy and reversing T cell exhaustion to a certain extent [20]. However, ICIs were only effective in a small number of patients, and even when ICIs work in patients, further T cell exhaustion occurred later in immunotherapy, or severe immune-related adverse events, leading to treatment failure [21–24]. In addition, in some cases, clinical efficacy might fall short of expectations due to the complex regulatory mechanisms of PD-1/PD-L1 or CTLA-4 in cancer immunity. These studies suggested that understanding the characteristics and causes of T cell failure and developing more effective, safer and cost-effective drugs are of great significance to ameliorate T cell exhaustion [25].

Due to its characteristics of multiple targets and multiple components, the application of traditional Chinese medicine (TCM) to improve human immunity has been widely used in clinical practice, especially in improving the immune function of tumor patients, with remarkable efficacy. Modern studies proved that TCM compounds and active ingredients improved T cell exhaustion to some extent by affecting T cells subsets in virus or tumor patients [26]. In this paper, we review the characteristics and causes of T cell exhaustion, and TCM targeting T cell exhaustion, providing a systematically reviewed the experimental progress of TCM regulating T cell exhaustion in infectious diseases and cancer in recent years. Although the current studies are mostly limited to animal and cell experiments, this review provides a comprehensive and valuable source of information for the development of subsequent experimental studies and clinical applications needed to further determine the role of TCM in improving the treatment of T cell exhaustion in infectious diseases and cancer.

## Characteristics of T cell exhaustion

T cell exhaustion is manifested by progressive loss of effector function. Exhausted T cells in infections and cancers often express high levels of IRs, including PD-1, CTLA-4, LAG-3, TIM-3, etc. These IRs are also known as immune checkpoints. Exhausted T cells can co-express multiple IRs. Thus, IRs can be used as a characteristic of T cell exhaustion. Recent studies have revealed the epigenetic and transcriptomic landscapes of T cell exhaustion, identifying several key depletion related molecules such as DNA methyltransferase 3 alpha (DNMT3A), thymocyte selection-associated high mobility group box (TOX), NR4a and Eomesodermin (Eomes). Disorders of depleted T cell metabolism have also been reported. These studies reveal patterns and mechanisms of T cell exhaustion, which may provide promising strategies for restoring T cell function. Strategies to reverse T cell exhaustion are as follows (Table 1).

**Table 1:** Strategies to reverse T cell exhaustion

Disease types	Intervention methods	Target	References
HIV	Anti-PD-L1	PD-1	[30]
HBV	Anti-PD-L1	PD-1	[31]
LCMV	Anti-PD-L1, anti-LAG-3	PD-1, LAG-3	[32, 33]
HIV	Knock-down of PD-L1 and PD-L2	PD-L1, PD-L2	[34]
HIV	Anti-CTLA-4	CTLA-4	[36]
HBV	Anti-CTLA-4	CTLA-4	[37]
Bladder cancer	Anti-CTLA-4	CTLA-4	[38]
LCMV	Anti-TIM-3	TIM-3	[39]
HCV	Anti-TIM-3	TIM-3	[42]
HIV	Anti-TIM-3	TIM-3	[43]
HCC	Anti-TIM-3	TIM-3	[45]
LCMV	Anti-LAG-3	LAG-3	[46]
RCC	Knock out of VISTA	VISTA	[49, 50]
HCV	Anti-PD-L1, anti-CTLA-4	PD-1, CTLA-4	[51]
HBV	Anti-PD-L1, anti-CTLA-4	PD-1, CTLA-4	[52]
LCMV	Anti-PD-L1, anti-TIM-3	PD-1, TIM-3	[53]
GC	Anti-PD-1, anti-PD-L1, anti-LAG-3, anti-TIM-3	PD-1, TIM-3, LAG-3	[54]
Osteosarcoma	Knockout of DNMT3A	DNMT3A	[56, 57]
LCMV	Knockout of TOX	TOX	[58]
Melanoma	Knockout of TOX	TOX	[59]
LCMV	Knockout of NR4A1	NR4A1	[61, 62]
LCMV	Knockout of Eomes	Eomes	[66]
LCMV	Knockout of T-bet	T-bet	[67]
CLL	BTK inhibitor	Glycolysis	[72]
LCMV	Anti-PD-L1	Glycolysis	[73]
Sarcoma	Anti-PD-1, anti-PD-L1, anti-CTLA-4	Glycolysis	[75]
NSCLC	Anti-PD-1	Glucose, lipid and mitochondrial metabolism	[76]
Melanoma	Knock down and overexpress of XBP1s	Cholesterol	[77, 78]
Lymphoma	Glutamine metabolism inhibitors	Glutamine metabolism	[79]
Breast cancer, cervical cancer	D-1MT	Tryptophan catabolism	[81]
HBV	Anti-CD3, and anti-CD28	Arginine	[82]
HIV	IL-15	Glycolysis	[84]
HCV	Histone methyltransferase inhibitors	Glycolytic and mitochondrial functions	[85]
Colon cancer, lymphoma, melanoma	DON, JHU083	Glutamine	[87]
HBV	Mitochondrion-targeted antioxidants	Mitochondria	[86]
ccRCC	Metformin	Mitochondrial ROS	[89]
Melanoma	Axitinib, anti-PD-1, anti-CTLA-4	Mitochondrial ROS	[91]
Melanoma, lung cancer, colorectal cancer	Overexpress of PGC1α	Mitochondrial metabolism	[92]
LCMV, melanoma	Knock out of VHL	HIF-1α, HIF-2α	[94]
Breast cancer	Knock out of HIF-1α	HIF-1α, VEGF-A	[95]

*RCC* renal cell carcinoma, *GC* gastric cancer, *CLL* chronic lymphocytic leukemia, *BTK* Bruton’s tyrosine kinase, *d-1MT* 1-methyl-d-tryptophan, *NSCLC* non-small-cell lung cancer, *IL-15* interleukin-15, *DON* 6-diazo-5-oxo-l-norleucine, *ccRCC* clear cell renal cell carcinoma

### Dysfunction and loss of function

In terms of effector function, the exhausted T cells show progressive dysfunction and even loss of function. T cells usually lose their ability to proliferate and kill first. During the intermediate stages of dysfunction, T cells lose other functions, such as the ability to produce TNF. Severe exhaustion of T cells eventually results in a partial or complete lack of ability to produce IFN⁃γ, β-chemokines or degranulation [27]. The function of the exhausted T cells is completely lost, ultimately resulting in the inability to eliminate the virus or tumor cells.

### The expression of inhibitory receptors

#### PD-1

Persistent high expression of inhibitory receptors and co-expression of multiple inhibitory receptors are important features of the immune response of exhausted T cells in chronic infection or cancer. PD-1 is one of the most widely studied inhibitory receptors in chronic viral infections and cancer. Its two known ligands are PD-L1 (B7-H1) and PD-L2 (B7-DC). PD-1 is mainly expressed on T cells, B cells, natural killer cell (NK), dendritic cell (DC) and macrophages, while PD-L1 is expressed on many cells, including tumor cells, B cells, T cells and macrophages, etc. The combination of PD-1 and PD-L1 can lead to T cell dysfunction. PD-1/PD-L1 immune checkpoints function in peripheral tissues and act as negative regulators of T cells to help control local inflammatory responses and maintain auto tolerance [28]. PD-1 on the surface of T cells is coupled with PD-L1 on the surface of tumor cells, resulting in Tyr phosphorylation in the ITSM domain in the cytoplasmic region of T cells. The phosphorylated Tyr can recruit SHP-1 and SHP-2 (Src homology region 1, 2, Src homology domains 1 and 2), SHP-2 dephosphorylates TCR-associated CD3ζ and zeta chain associated protein kinase 70 (ZAP70), inhibits downstream signaling and interferes with IL-2 secretion. The activation of immune cells by TCR/CD28 signals is weakened, inhibiting T cell proliferation and cytokine secretion [29]. Inhibition of PD-1 induced HIV or HBV-specific CD8 T cells to produce cytokines, such as IFN-γ, enhancing the immune response [30, 31]. A comparison of the gene expression profiles of virus-specific CD8 T cells from acute or chronic lymphocytic choriomeningitis virus (LCMV)-infected mice showed that the inhibitory receptor PD-1 was highly expressed in chronic infection [32], and was associated with an exhausted CD8 T cells phenotype [33]. In HIV infection, by blocking PD-L1, T cells regained their ability to secrete cytokines IFN-γ, IL-2, and IL-12 after restimulation [34]. In addition, blocking PD-1/PD-L1 partially improved the effector function of exhausted T cells in vivo [5].

#### CTLA-4

In addition to PD-1, exhausted T cells expressed a lot of inhibitory receptors, including CTLA-4, TIM-3, LAG-3, and VISTA. Although individual expression of PD-1 or its inhibitory receptors was not sufficient to indicate T cell exhaustion, co-expression of multiple inhibitory receptors was thought to be a major feature of T cell exhaustion [35]. CTLA-4 was found to be associated with T cell exhaustion. Studies indicated that high expression of CTLA-4 affected the quality of T cell response function after infection with HIV [36] and HBV [37]. The high expression of CTLA-4 also weakened T cell function in cancer [38].

#### TIM-3

In addition, high expression of TIM-3 on effector T cells also indicated severe T cells failure or dysfunction [39–41]. TIM-3 frequency and expression levels were positively correlated with T cell exhaustion status and infection severity in HIV, HCV, and LCMV infections [39, 42, 43]. High expression of TIM-3 also led to T-cell exhaustion [44]. Moreover, TIM-3 was significantly up-regulated in NK in HCC, and inhibited its cytokine production and cytotoxic activity by inhibiting phosphatidylinositol-3-kinase (PI3K)/Akt and the mammalian target of rapamycin (mTOR)1 signaling pathway, leading to dysfunction of NK cells in hepatocellular carcinoma (HCC) [45].

#### LAG-3

In addition to these IRs, a growing number of studies have linked the expression of inhibitory receptors such as LAG-3 and VISTA to T cell exhaustion in chronic infections and some cancers. The expression of LAG-3 was positively correlated with T cell exhaustion status and infection severity in LCMV infections [46]. A study showed that LAG-3 activation enhanced intra-tumoral Tregs activity, blocking improved T cells function and reactivating tumor-infiltrating CD8^+^ T cells (CD8^+^TIL) to eliminate tumor cells [47].

#### VISTA

As a ligand of VISTA, V-Set and immunoglobulin domain containing 3 (VSIG-3) blocked the production of cytokines and chemokines [48]. The expression of VISTA or VSIG-3 was elevated in many cancers. Studies found that VISTA was a regulatory immune checkpoint. The blockade of VISTA/VSIG-3 could be used as a new target for immune checkpoint therapy. Spontaneous T cell activation occurred in VISTA-deficient mice [49]. Moreover, VISTA was associated with T cell exhaustion marker TOX, and VISTA positive was associated with a poorer prognosis in clear cell renal cell carcinoma (ccRCC) [50].

In addition, exhausted CD8^+^T cells expressed multiple IRs. The co-expression pattern of IRs affected the function of T cells during chronic infection, and the severity of chronic infection was related to the number and intensity of IRs expressed. In general, the more IRs exhausted T cells co-expresses, the more severe its exhaustion state [33]. Studies showed that the effector function of patient-specific CD8 T cells was restored by co-inhibition of PD-1 and CTLA-4 expression in chronic HCV [51] or HBV infection [52]. Studies showed that CD8^+^T cells that co-expression of TIM-3 and PD-1(TIM-3^+^PD-1^+^) had more severe exhaustion than those that only express PD-1(TIM-3^−^PD-1^+^). Compared with blocking either pathway alone, the blocking of TIM-3 and PD-1 pathways at the same time had a synergistic effect in restoring the antiviral immunity of T cells [53] and tumor inhibition [54]. Similarly, studies found that blocking LAG-3 alone had a minimal effect on the reversal of CD8^+^T cell exhaustion, but the combined blocking of LAG-3 and PD-1 collaboratively improved CD8^+^T cell response and significantly reduced viral load in vivo [33]. These studies suggested that co-expression of multiple IRs might play a synergistic role in T cell exhaustion.

### Changes in the epigenetic and transcriptional landscape

T cell exhaustion results in extensive changes in transcription profile and chromatin. After chronic LCMV infection, the accessibility of T cell chromatin in exhausted T cells showed that exhausted T cells exhibited different chromatin phenotypes from effector T cells and memory T cells. These findings suggested that exhaustion was a specific T cell fate associated with distinct changes in chromatin phenotype [55]. DNA methylation induced by DNMT3A has been shown to play a key role in chromatin accessibility and T-cell exhaustion, and the tumor killing effect of chimeric antigen receptor T-cell immunotherapy (CAR-T) after DNMT3A knockout is significantly enhanced [56, 57]. Furthermore, studies showed that DNA-binding protein TOX affected the survival and development of T cell exhaustion. It was involved in epigenetic remodeling during T cell exhaustion in chronic viral infections and cancer [58, 59]. Moreover, TOX deletion of virus-specific CD8^+^ T cells did not prevent the formation of effector T cells and memory T cells during acute LCMV infection. However, in the case of chronic LCMV infections and cancer, TOX deficiencies had improved antiviral and anti-tumor activity, and were associated with decreased expression of CD8^+^ T cells suppressor receptors. However, CD8^+^ T cells with TOX deficiency did not persist during chronic viral infection and cancer development. This suggested that TOX mediated signaling and epigenetic programming sustained the survival of exhausted T cells in the presence of prolonged exposure to antigens [60].

In addition to DNMT3A and TOX, nuclear receptor transcription factor NR4a drove T cell exhaustion by coordinating epigenetic and transcriptomic changes associated with exhaustion in CD8^+^ TILs and CAR-T cells. In adoptive cell transfer of tumor-specific CD8^+^ T cells and CAR-T cells, the absence of NR4a increased the antitumor activity of T cells [61, 62]. Moreover, in CD8^+^ TILs, interactions between TOX and NR4a might further enhance phenotypic and epigenetic expression of exhausted T cells.

Furthermore, studies showed that Eomes and T-bet were transcription factors that regulated T cells differentiation [63, 64]. And up-regulation of Eomes was necessary for the formation of long-term memory-like cytotoxic T cells [65]. Loss of Eomes led to defects in memory T-cell populations [66]. Conversely, T-bet induced the production of inhibitory receptor molecules, such as PD-1 and TIGIT [67]. Exhausted CD8^+^ T (Tex) cells contain two subpopulations: Tex progenitor cells and terminal Tex cells. Tex progenitor cells highly expressed T-bet (T-bet^Hi^ PD-1^Int^), regenerated by division and maintain virus specificity. However, they differentiated to express Eomes (Eomes^Hi^PD-1^Hi^). This subgroup was more effective against the virus, but cannot self-replicate [27, 68, 69].

An additional consideration was that the exhausted epigenetic state was largely irreversible. Analysis of chromatin accessibility in HCV and HIV-specific responses identified a core epigenetic program for CD8^+^ T cell exhaustion that undergone only limited remodeling before and after resolution of infection [70]. In cancer therapy, ICIs could often transiently reprogram or activate T cells to restore effector cell-like transcriptional and functional phenotypes. However, under the influence of DNA methylation and histone modification, the chromatin state of T cells was still largely stable, which could quickly disappear the effect of ICIs and T cells return to exhaustion. This regulatory mechanism was also referred to as “epigenetic traces”. Collectively, these findings demonstrated that epigenetic alterations played an important role in orchestrating T-cell exhaustion.

### Metabolic reprogramming

#### Carbohydrate metabolism

Studies indicated that histone acetylation required acetyl-CoA, which was produced by the catabolism of acetate, citrate or pyruvate. Limited glucose availability or glycolytic metabolism might affect chromatin accessibility and further hinder T cells immunity. Lactate dehydrogenase A (LDHA) depletion decreased the acetylation of histone H3 lysine 9 residue (H3K9) at the *Ifng* site in mouse CD4^+^ T cells by inhibiting aerobic glycolysis, resulting in decreased acetyl-CoA levels. Acetate supplementation promoted histone acetylation and chromatin accessibility, restoring IFN-γ expression in CD8^+^ T cells under glucose limitation [71]. Studies showed that immune checkpoints inhibited T cells function by inhibiting glycolysis in immune cells, and ICIs restored glucose uptake by immune cells [72]. For example, the interaction of PD-1 with its ligand PD-L1 regulated early glycolysis and mitochondrial changes and inhibited the transcriptional coactivator PGC-1α. Overexpression of PGC-1α reversed the function of exhausted CD8 T cells by improving biological energy, and treated with anti-PD-L1 could reprogram the metabolism of a portion of exhausted CD8 T cells [73]. In addition, impaired glycolysis and reduced metabolic responses was also found in HBV, HCV, or HIV infected patients, as well as in LCMV infected mice. Although the microenvironment of viral infection was quite different from that of TME, PD-1 signaling prevented aerobic glycolysis, which led to metabolic impairment during chronic LCMV infection. Thus, chronic viral infection led to similar impairment of T cells metabolism by promoting glucose deprivation and pseudohypoxia [74]. A study also found that increasing the glucose uptake and utilization ability of T cells was beneficial to the activation of T cells in cancer [75].

#### Lipid metabolism

Exhausted T cells from both chronic viral infection and cancer exhibited metabolic abnormalities, and lipid metabolism contributed to CD8^+^T cell exhaustion. PD-1^hi^ CD8^+^ TILs had higher lipid content than PD-1^lo^ CD8^+^ TILs in non-small cell lung cancer patients. Abnormal lipid metabolism promoted T cell exhaustion, and the promotion of lipid catabolism effectively enhanced the effector function and tumor killing activity of CD8^+^ TILs [76, 77]. Moreover, due to the accumulation of adipocytes in tumors and the up-regulation of fatty acid synthesis in cancer cells, cholesterol and other lipids in tumor microenvironment (TME) caused T cell exhaustion through the perturbation of ER stress response and ferroapoptosis in CD8^+^ TILs [74]. Although little is known about the role of cholesterol metabolism in chronic infection, it is widely accepted that cholesterol metabolism is critical in T-cell function and cancer failure. Studies showed that cholesterol accumulation promoted the expression of inhibitory receptors such as PD-1 by increasing ER stress, leading to CD8^+^ T cell exhaustion. Inhibition of ER stress sensor XBP1 or cholesterol reduction could effectively restore effector T cells function [77, 78].

#### Amino acid metabolism

Glutamine played an important role in the rapid proliferation of CD8^+^T cells, especially in effector CD8^+^T cells with highly active glutamine metabolism. Effector T cells consumed glutamine as a non-glucose fuel for mitochondrial oxidation and retained glucose for the production of macromolecules required for rapid proliferation. Glutamine deficiency increased the expression of inhibitory receptors such as PD-1 and LAG-3, leading to CD8^+^ T cell exhaustion [79]. Since arginase and indoleamine-pyrrole 2,3-dioxygenase (IDO) were highly expressed in DCs, myeloid-derived suppressor cells (MDSCs), and tumor-associated macrophages (TAMs), the absence of amino acids such as arginine and tryptophan could further impair metabolic fitness and alter the activation and differentiation of TILs [80]. Arginine depletion promoted a decrease in T-cell proliferation, cytokine production, and TCR expression. The high activity of IDO consumed tryptophan in the TME and impaired the activity of mTOR through the activation of the kinase GCN2, leading to T cells dysfunction [81]. In addition, kynurenine produced by tryptophan degradation inhibited T cells immunity by activating aromatic hydroxyl receptors [82]. T cells activation was also required large amounts of methionine, which acted as a donor of methyl groups during cellular methylation, affecting epigenetic reprogramming of T cells differentiation [83]. Studies indicated that viral antigen-specific exhausted CD8^+^ T cells exhibited decreased metabolic fitness in patients infected with HBV, HCV, or HIV [84–86]. Blocking the tumor metabolism of glutamine not only reduced hypoxia, acidosis and nutrient consumption in the tumor microenvironment, but also promoted T cell activation and prolong the life of T cells [87]. These studies showed that metabolic reprogramming influenced T cell exhaustion.

#### Mitochondrial fitness

As a key regulator of energy, current studies confirmed that the importance of mitochondria in coordinating the function of CD8^+^T cells and their importance in the generation of CD8^+^T cell exhaustion. Mitochondria were key organelles that regulated cell metabolism and activity [88]. In chronic renal LCMV-infected mice, early activated LCMV-specific CD8^+^ T cells contained depolarized mitochondria that impede T cell metabolic adaptation [73]. In HBV infection, after depletion of CD8^+^ T cells, the expression of genes encoding mitochondrial proteins and regulators of mitochondrial biogenesis was also greatly decreased and contained more depolarized mitochondria [86]. The accumulation of depolarized mitochondria led to the production of mitochondrial reactive oxygen species (mtROS). Exhaustion of mtROS effectively restored the effector and antiviral response of HBV-specific exhausted T cells [89]. Thus, targeted clearance of mtROS and improvement of depolarized mitochondria may prevent T cells from differentiating into a state of terminal depletion and maintain their reactivity to ICIs therapy [90]. Peroxisome proliferator-activated receptor-γ coactivator 1α (PGC-1α) acted as a transcriptional coactivator to promote the function of CD8^+^TILs by coordinating mitochondrial biogenesis and antioxidant activity [91]. In early activated LCMV viral infection, over-expression of PGC1α in specific CD8^+^ T cells reduced the exhaustion phenotype and alleviated mitochondrial dysfunction, improving metabolic adaptation [92]. Blymphocyte-induced maturation protein 1 (Blimp-1), a transcriptional repressor, was highly expressed in terminally exhausted CD8^+^T cells in the B16 melanoma model. Blimp-1 also accelerated the exhaustion of CD8^+^T cells by inhibiting the metabolic changes of PGC-1α [92].

#### Hypoxia

Hypoxia is another key factor that dampens T-cell function in response to oxygen deprivation and pseudohypoxia in the context of chronic infection and tumors [93]. Hypoxia has a unique two-sided effect on immune cell function. Hypoxia-inducible factor (HIF), a key factor in the hypoxic response, was activated in response to hypoxia and inactivation of proline hydroxylase. HIF signaling pathway played an important role in the induction of glycolysis in chronic LCMV infection and melanoma. Moreover, HIF activation during chronic infection led to over-activation of T cells [94], while the absence of HIF in T cells impaired the activity of tumor-induced T cells and led to a reduced T cell activation phenotype in lung cancer and melanoma [95]. In mouse breast cancer, HIF-1α deficiency reduced tumor infiltration and cytotoxic function of CD8^+^T cells [95].

## Factors related to the occurrence and development of T cell exhaustion

T cell exhaustion is influenced by a variety of factors, the level and duration of antigen persistence, inhibitory and promotive cytokines, and immunomodulatory cells.

### The level and duration of antigenic stimulation

A key feature of chronic infection or cancer was constant exposure of T cells to antigens, with both high antigen load and prolonged antigen exposure, leading to more severe T cell exhaustion. During more protracted infections, effector T-cell numbers and the functional quality of the response generally decrease, with a further exhaustion phenotype developing as the viral load remains high. Mice infected with chronic strains of LCMV differentiated into fully CD8^+^ memory T cells if they were isolated from sustained antigenic stimulation early (about 1 week after infection). However, these T cells were exposed to persistent antigens for 2–4 weeks, T cells became exhausted and failed to resume normal memory differentiation [96]. In HIV, persistent antigen exposure (or co-stimulation) in the absence of IL-1β results in systemic CD4^+^ T-cell exhaustion. In this context, the deleterious effects of HIV infection on CD4^+^ bone marrow cells, including APCs, capable of producing IL-1β, might lead to altered T cell lineage homeostasis and reduced cell division [97]. These studies suggested that chronic antigenic stimulation and its duration might be a key factor in T cell exhaustion.

### Cytokines

Similar to the inhibitory receptors described above, cytokines affect the function and status of T cells in chronic infections and tumors. Cytokines that promoted T cell exhaustion included IL-10, transforming growth factor-beta (TGF-β), IL-6 and tumor necrosis factor-alpha (TNF-α), as well as chemokines. IL-10 and TGF-β promoted PD-1 expression on the surface of T cells and were involved in the induction of T cell exhaustion and viral persistence [98–101]. Studies showed that the expression of IL-10 was upregulated during persistent infection of several viruses, including HBV, HCV, HIV, and LCMV. Blocking the expression of IL-10 enhanced viral control and improved responses to exhausted T cells [102–105]. Blocking T cell acceptance of TGF-β signaling during chronic LCMV infection improved CD8^+^T cell function and prevented severe exhaustion of CD8^+^T cells [106]. The presence of IL-10 and TGF-β was also found to inhibit T cells function in cancer [107, 108]. Studies also found that other immunomodulatory cytokines such as IL-6 and TNF-α also played a role in CD8 T cell exhaustion in chronic infection and cancer. Inhibition of these molecules or the receptors that bind to them serves as a potential strategy for reversing T cell exhaustion [2]. Studies showed that IL-6 promoted T cell exhaustion through IL-6/STAT3/PD-1 transcriptional regulation [98], TNF-α induced PD-L1 expression and attenuated immune cell activation, and blockade of TNF-α also reversed T cell exhaustion [109]. Studies found that in COVID-19, it did not directly attack T cells, but triggered the release of cytokines, which in turn drove the exhaustion of T cells. CD4^+^ and CD8^+^ T cells were significantly decreased in COVID-19 patients, the number of T cells was negatively correlated with serum IL-6, IL-10 and TNF-α concentrations. The level of PD-1, a marker of T cell exhaustion, was significantly increased [110]. In addition, cytokine storm promoted T cell exhaustion through the cytokine-receptor axis. Blocking the cytokine receptor axis, such as CCL2-CCR2, CCL3-CCR1, CCL3-CCR5, CCL4-CCR5, CCL3L1-VSIR, and CCL3L1-CCR1, might be a new strategy to reverse T cell exhaustion and further treat the immune failure associated with severe coronavirus pneumonia with cytokine storm [111].

Cytokines that antagonized T cell exhaustion included IL-2 and IL-12. IL-2, known as T cells growth factor, was essential for T cell proliferation and survival and for the formation of effector and memory cells [112]. By IL-2 supply, defective virus-specific CD8 T cells restored the proliferation and differentiation capacity of IFN-γ-producing cells [113]. However, IL-2 played a bidirectional role in the regulation of T cells in tumors. In the early stage of tumor, IL-2 induced the differentiation of effector CD8^+^T cells in an autocrine manner, and promoted the activation and proliferation of CD8^+^T cells [114]. However, in the late stage of tumor, IL-2 induced CD8^+^ T cell exhaustion in the tumor microenvironment by activating STAT5-5-HTP-AhR pathway [115]. IL-12 was produced by activated antigen-presenting cells (APC). It promoted the development of Th1 responses and was a potent inducer of IFN-γ production by T cells and NK cells. IL-12 induced NK cell and T cells proliferation, enhancing cytotoxic mediator expression and cytokine production, especially IFN-γ [114]. Moreover, IL-12 alone could effectively restore the secretion of multiple cytokines and the cytotoxic activity of HBV-specific CD8 T cells. In addition, IL-12 restored IFN-γ production in HBV-specific CD8 T cells [116], but the effects of different cytokines on exhausted T cells needed to be further confirmed.

### Other immune cells

In chronic infection, CD4^+^T cells played an important role in regulating the response of CD8^+^T cells. CD4^+^T cells activated antigen-presenting cells through CD40 and CD40 ligand interaction to secrete chemokines and cytokines. At the same time, CD4^+^T cells produced IL-2 and IL-21 cytokines that directly acted on CD8^+^T cells [117, 118]. Short-term depletion of CD4^+^T cells in mice chronically infected with HIV led to high viral load and severe exhaustion of CD8^+^T cells [119]. A study showed that in LCMV mice, effective adjuvant action of CD4^+^T cells with initial and antigen-specific effects could reverse exhaustion of CD8^+^T cells in vivo [120]. In addition, CD8^+^T cell exhaustion status was also influenced by NK cells, which produced IL-10 and TGF-β to inhibit T cell activation and led to T cell exhaustion by impacting APC function during persistent viral infection. NK cell exhaustion significantly reduced the expression of inhibitory receptors such as PD-1 and LAG-3 in T cell [121, 122].

Other immune regulatory cells such as regulatory T cells (Tregs), MDSCs, M2 macrophages are frequently increased in infections and tumors [27]. Treg cells often abnormally accumulated in TME and expressed immunosuppressive molecules, including the cytokines IL-10, IL-35, and TGF-β, which suppressed the anti-tumor effects of T cells [123–125]. In addition, studies showed that intra-tumoral Treg cells promoted T-cell exhaustion in CD8^+^TILs through interactions with IL-10 and IL-35 in melanoma [125]. Large accumulation of TAMs impaired T cell-mediated anti-tumor responses and promoted T cell exhaustion due to IL-10 production and PD-L1 expression [60]. MDSCs suppressed the innate immune system mainly by secreting IL-10 and TGF-β, which induced macrophages to develop an immunosuppressive M2 phenotype and negatively affected NK cells maturation and DC function [126]. MDSCs promoted the expression of PD-1 [127, 128], PD-L1, LAG3, CTLA-4 and TIM-3 on CD4 cells, and partially induced the exhaustion of activated T cells through IFN-γ [129].

The occurrence and development of T cell exhaustion was a complex process, which was influenced by many factors. Changes in any aspect of antigenic stimulation, cytokines or immune cells might lead to poor response to chronic infections or cancer, further promoting the development of a state of exhaustion.

## Traditional Chinese medicine and T cell exhaustion

### Traditional Chinese medicine compound

#### Bushen Jianpi recipe

TCM compounds played an important role in the treatment of infectious diseases and cancer (Table 2). Studies also found that Bushen Jianpi Recipe intervention in a mouse model of T cell exhaustion induced by continuous stimulation of purified protein derivative (PPD) significantly restored the number of CD4^+^ and CD8^+^T cells in the spleen, and significantly reduced the expression of PD-1 on CD4^+^T cells. Moreover, the levels of IL-2 and IFN-γ secreted by splenic lymphocytes were significantly increased, and the ability of anti-infection of mice was significantly enhanced [130].

**Table 2:** The effect of TCM compounds and active components on the regulation mechanism of T cell exhaustion

Drugs	Disease type	Mechanism of effect	Cell type/model	References
Bushen Jianpi Recipe	Mycobacterium bovis BCG purified protein derivative	Enhances IFN-γ and IL-2 expression, up-regulates CD4^+^ and CD8^+^ T cells and inhibits PD-1 expression level on CD4^+^ T cells	Mice in vivo model	[130]
Dahuang Fuzi Baijiang Decoction	Colorectal cancer	Inhibits the PD-1^hi^TIM^3+^ subset and CCL2 expression and enhances the PD-1^int^TCF^+^ population	MC38 cell line and mice in vivo model	[131]
Yangyin Fuzheng Jiedu Prescription	Hepatocellular carcinoma	Enhances the ratio of CD3^+^ and CD8^+^ T cells in the peripheral blood, spleen, and tumor tissue and the expression of TNF-α, and IFN-γ and down-regulates the expression of PD-1, TIGIT, TIM-3 in CD8^+^ T cells and IL-1β, IL-6, and IL-10 in the serum and tumor tissues	Mouse HCC cell line H22 and mice in vivo model	[132]
ShenQi FuZheng Injection	Lung cancer-related fatigue	Inhibits PD-L1, TIM-3 and FOXP3 and IL-2, IFN-γ and TNF-α expression in serum	Lewis Lung carcinoma and mice in vivo model	[133]
Yangyin Fuzheng Decoction	Lung cancer	Inhibits the expression of PD-1^+^ CD8^+^ T cells and PD-1, PD-L1, TIM-3 in tumor tissues and increases PD-1^−^CD8^+^ T cells in peripheral blood and CD4^+^CD25^+^ FoxP^+^ T cell in tumor tissues	PD-L1^+^Lewis and Lewis lung cancer cells and mice in vivo model	[134, 135]
Qiyusanlong Decoction	Lung cancer	Increases TNF-α, IL-1, IL-12 and inhibits CXCL-9, CCL-17, STAT6 and MTOR expression	Lewis lung cancer cells and mice in vivo model	[138]
Compound kushen injection	Hepatocellular carcinoma	Reduces the distribution and polarization of M2-TAM, promotes M1-TAM	Hepa1-6 or LPC-H12 cell lines and mice in vivo model	[139]
Astragalus membranaceus polysaccharide	Breast cancer	Enhances IFN-γ and IL-2 expression	4T1 breast tumor cell line and mice in vivo model	[144]
Dihydroartemisinin	Hepatocellular carcinoma	Promotes CD4^+^ T cell infiltration in spleen and CD8^+^ T cell infiltration in tumor tissue	HepG2215 cell line and mice in vivo model	[145]
Extract derived from the sporoderm-breaking spores of *G. lucidum*	Breast cancer	Increases cytotoxic T cell (Tc) population and the ratio of Tc to helper T cell (Th) and decreases PD-1 and CTLA-4 expression	Breast cancer 4T1-cell line and mice in vivo model	[146]
Hirsutella sinensis fungus	Breast cancer	Increases CD44^Low^CD62L^Hi^ and CD44^Hi^CD62L^Low^ populations in the tumor-infiltrating CD8^+^ T cells and enhances IFN-γ and granzyme B and reduces PD-1, TIGIT, CTLA-4 expression	4T1-Luc cells and mice in vivo model	[147]
Pinellia pedatisecta Schott extract	Cervical cancer	Up-regulates the expression of MHCII, CD80, CD86, IL-12 and promotes CD4^+^ and CD8^+^ T cells and induces the differentiation of IFN-γ^+^CD4^+^ and GZMB^+^CD8^+^ T cells	TC-1 tumor cell line and mice in vivo model	[151, 152]
Astragalus membranaceus and extract	Renal cell carcinoma	Increases IL-21 expression	Renal cell carcinoma patients	[153]
Lycium barbarum polysaccharide	Liver cancer	Increases CD8^+^ T cells and decreases TGF-β and IL-10 expression	Mouse HCC cell line H22 and mice in vivo model	[154]
Luteolin and its derivative apigenin	Lung cancer	Inhibition of STAT3 phosphorylation, down-regulates IFN-γ-induced PD-L1 expression and increases CD8^+^ T cells, IFN-γ, TNF-α and Granzyme B	KRAS-mutation NSCLC (H460, H358and A549) cells and mice in vivo model	[155]
Bisdemethoxycurcumin	Bladder cancer	Enhances IFN-γ, granzyme B, and perforin expression	MB49 cells and mice in vivo model	[156]

#### Dahuang Fuzi Baijiang decoction

Moreover, studies found that obesity increased the occurrence of colorectal cancer, and it led to T cell exhaustion by increasing PD-1 expression and reducing IFN-γ and TNF-α production in TILs. PD- 1^hi^TIM3^+^showed a defect in IFN-γ production and a residual capacity in TNF-ɑ production. The PD-1^hi^TIM3^+^ subset was typically defined as terminally differentiated, exhausted T cells, whereas the co-existence of intermediate expressed PD-1 (PD-1^int^) and TCF-1 gave Tex stem cell properties. Dahuang Fuzi Baijiang Decoction (DFB) enhanced the efficacy of PD-1 checkpoint blockade by restricting the PD-1^hi^TIM3^+^ subset and amplifying the PD-1^int^ TCF^+^ to suppress tumor growth in high-fat diet-induced obese mice. The recombinant chemokine C–C-motif receptor 2 (CCR2) ^+^CD8^+^ subset was defined as Tex terminals and differentiation from progenitor cells to Tex terminals was driven, at least in part, by the chemokine (C–C motif) ligand 2 (CCL2)/CCR2 axis, CCR2 inhibitors enhanced the response to anti-PD-1 by promoting Tex progenitor cell counts. In conclusion, DFB inhibited CCL2 and retained progenitor Tex to suppress CRC progression in the obese microenvironment [131].

#### Yangyin Fuzheng Jiedu prescription

TCM compounds also play an important role in improving T cell exhaustion in a variety of cancers. Studies indicated that Yangyin Fuzheng Jiedu Prescription (YFJP) effectively inhibited solid tumor growth and spleen index in mice with liver cancer. In addition, YFJP maintained high percentages of CD3^+^ and CD8^+^ T cells in peripheral blood, spleen and tumor tissues, while reducing the expression of multiple inhibitory receptors on CD8^+^ T cells. These included PD-1, TIGIT and TIM-3. In addition, YFJP significantly reduced the expression of inflammatory and immunosuppressive cytokines, including IL-1β, IL-6 and IL-10, while increased the expression of cellular effectors TNF-α and IFN-γ in serum and tumor tissues. It also restored the cytotoxicity of tumor-infiltrating T cells. It has also been found that PD-1^+^TIM-3^+^ double positive T cells have a more sensitive and effective prognostic value than PD-1^+^ or TIM-3^+^CD8^+^ T cells. Surprisingly, PD-1/TIM-3 double-positive T cells were significantly reduced in peripheral blood and tumor tissue in the YFJP group [132].

#### ShenQi FuZheng injection

In addition, Chinese Medicinal Preparations also played an important role in the treatment of T cell exhaustion. Chinese Medicinal Preparations increased the number of CD4^+^ T cells, CD8^+^ T cells and the ratio of CD4^+^/CD8^+^, regulating the body's immune function and enhancing the immune response. Studies also found that ShenQi FuZheng Injection (SFI) inhibited the growth of lung cancer in mice and reduced the depression-like behavior of tumor-bearing mice. The level of CD19 protein and CD4/CD8 were increased in SFI group, indicating that SFI could enhance the immune function of tumor-bearing mice. SFI also significantly down-regulated the expression levels of PD-L1, TIM-3 and FOXP3 in tumors and decreased the expression of FOXP3 in spleen. In addition, SFI reduced the levels of proinflammatory cytokines IL-2, IFN-γ, and TNF-α in spleen, However, the mRNA levels of IL-2, IFN-γ and TNF-α in tumor tissues were significantly increased, while IL-6 mRNA levels were significantly decreased. These results suggested that SFI improved symptoms of fatigue, alleviated dysfunction of T cell exhaustion by inhibiting proinflammatory cytokines produced by peripheral immune cells, and enhanced antitumor immunity through PD-L1, TIM-3, and FOXP3 [133].

#### Yangyin Fuzheng decoction

In lung cancer, Yangyin Fuzheng Decoction promoted the secretion of IL-2 and IFN-γ by splenic lymphocytes, inhibited the secretion of IL-10 and TGF-β by splenic lymphocytes. Moreover, it also reduced the number of PD-L1^+^CD8^+^T cells in peripheral blood and increased the number of PD-L1^−^CD8^+^T cells. It also reduced the expression of CD4^+^CD25^+^FoxP^+^T, PD-L1, PD-1, TIM-3 protein in tumor tissues and the expression of PD-L1 and PD-1 mRNA in tumor tissues, increasing the effect of atezolizumab on the proliferation of lung cancer cells [134, 135].

#### Qiyusanlong decoction

Moreover, studies also indicated that Qiyusanlong Decoction (QYSL) significantly promoted CD8 T cell activation in lung cancer. QYSL promoted the polarization of M1 macrophages and inhibited tumor growth by promoting the expression of TNF-α, IL-1, and IL-12. Studies showed that M2 macrophages inhibited the activation of effector T cells by promoting the high expression of CCL17. Furthermore, M2 macrophages also inhibited CD8^+^ T cell infiltration by decreasing the expression of CXCL9 [136, 137]. A study found that M2 macrophages were significantly increased in tumors. QYSL could inhibit the tumor growth by down-regulating the expression of M2 macrophage-related genes CXCL-9 and CCL-17 and inhibiting the activation of M2 macrophages. STAT6 and MTOR played important roles in the polarization of M2 macrophages. QYSL inhibited the expression of STA6 and MTOR and effectively inhibited the polarization of M2 macrophages. Collectively, QYSL suppressed tumor growth by inhibiting M2 macrophage polarization and modulating the tumor immune microenvironment [138].

#### Compound kushen injection

A study showed that compound kushen injection (CKI) activated the pro-inflammatory response of tumor-associated macrophages in the HCC microenvironment and reduced immunosuppression through triggering tumor necrosis factor receptor superfamily member 1 (TNFR1)-mediated cascade of NF-κB and p38 MAPK signaling. CKI also reduced the distribution and polarization of M2-TAM, promoted M1-TAM, and significantly promoted the proliferation and cytotoxic ability of CD8^+^ T cells, thus leading to the apoptosis of HCC cells [139].

### Active ingredients of traditional Chinese medicine

The active ingredients of traditional Chinese medicine also play an important role in restoring T cell exhaustion (Table 2).

#### Polyphenolic compounds

Polyphenolic compounds significantly regulated PD-L1 expression through a variety of pathways and increased gene transcription by targeting epigenetic processes, affecting the stability of transcription factors or mRNA. In addition, polyphenolic compounds affected the production and structure of PD-L1 through post-translational modification processes, such as ubiquitination and glycosylation. It also blocked the PD-1/PD-L1 pathway by directly blocking or increasing PD-L1 degradation, activating exhausted T cells and anti-tumor immunity [140, 141]. Studies indicated that polyphenolic compounds modulated TIL cell subsets by cytokine secretion, increasing the proportion of CD4^+^CD8^+^ TILs, and enhancing the activity of CD8^+^ CTLs in TME [142, 143]. These studies suggested that TCM had immunomodulatory effects and improved the state of T cell exhaustion in infectious diseases and cancer to a certain extent, which was beneficial for anti-infection and anti-tumor treatment.

#### Astragalus membranaceus polysaccharide

Furthermore, *Astragalus membranaceus* polysaccharide (APS), one of the active components of *Astragalus membranaceus*, promoted the expression of IL-2 and IFN-γ in the serum of mice, and induced low dose anti-PD-1 response. And it could delay the occurrence and development of 4T1 breast cancer by increasing the activity of T cells. Anti-PD-1 produced by APS stimulation. The isolated single strand fragment variable (scFv) S12 had the highest binding affinity with PD-1 (20 nM), which completed the interaction between PD-1 and PD-L1, and blocked the effect of PD-L1 inducing T cell failure in peripheral blood monocytes in vitro [144].

#### Dihydroartemisinin

The efficacy of anti-PD-1 therapy in HCC is not as expected. YAP1 was highly expressed and activated in liver cancer tissues. The mechanism of YAP1 in immune escape of liver cancer remains unclear. Anti-PD-1 treatment increased YAP1 expression in liver cancer cells and increased the expression of exhausted CD4^+^ and CD8^+^ T cells in blood and spleen of liver tumor mice. Knockdown of YAP1 inhibited the expression of PD-L1 and promoted the proportion of CD4^+^ and CD8^+^ T cells in liver tumor microenvironment. Dihydroartemisinin (DHA) inhibited YAP1 expression and broke immune escape in liver tumor microenvironment, which was manifested by decreased PD-L1 and increased infiltration of CD8^+^ T cells in liver tumor cells. DHA combined with anti-PD-1 treatment promoted CD4^+^ T cell infiltration in spleen and CD8^+^ T cell infiltration in tumor tissue. This suggests that DHA improved the effect of anti-PD-1 [145].

#### Extract derived from the sporoderm-breaking spores of *G. lucidum*

In breast cancer, studies showed that extract derived from the sporoderm-breaking spores of *G. lucidum* (ESG) inhibited the growth of 4T1 in vivo, and significantly increased the number of cytotoxic T cells (Tc) and the ratio of Tc to helper T cells (Th) in peripheral blood. A similar Tc promotion was found in tumor-infiltrating lymphocytes. In addition, ESG significantly reduced the expression levels of PD-1 and CTLA-4 in spleen and tumors, indicating that ESG effectively restored T cells function by inhibiting the co-inhibitory checkpoint to restore the exhaustion state [146].

#### Hirsutella sinensis fungus

In breast cancer, *hirsutella sinensis* fungus (HSF) inhibited tumor growth and lung metastasis of mice, decreased the expression of inhibitory receptors such as PD-1, TIGIT and CTLA-4, and suppressed the percentages of PD-1^Int^ and PD-1^Hi^CD8^+^T populations. Furthermore, the PD-1^Int^CD8^+^T cell population highly expressed T-bet both in vivo and in vitro, HSF inhibited T-bet expression in CD8 cells as well as PD-1^Lo^ and PD-1^Hi^cell populations. In addition, Eomes was highly expressed in PD-1^Int^ and PD-1^Hi^ cell populations, HSF promoted Eomes expression in CD8 T cells, PD-1^Lo^ PD-1^Int^ and PD-1^Hi^ cell populations. These findings suggested that HSF improved T cell function mainly by promoting the expression of Eomes [147].

#### Pinellia pedatisecta Schott extract

Immune cells played a key role in initiating, stimulating and maintaining immune responses against tumor progression [148]. DCs have dual effects on immune stimulation or suppression [149]. Immature DCs expressed low MHC and costimulatory molecules such as CD40, CD80, CD83 and CD86, maintaining immune tolerance by inducing T cells loss or anergy and inhibiting T cell differentiation [148, 150]. Upon maturation, DCs had high expression of major histocompatibility complex class II (MHCII) and costimulatory molecules and stable production of IL-12, which further promoted CTL and Th1 responses. Pinellia pedatisecta Schott extract (PE) promoted the expression of MHCII, CD80, CD86 and IL-12, the proliferation of CD4^+^ and CD8^+^ T cells, and induced the differentiation of IFN-y^+^CD4^+^ and GZMB^+^CD8^+^ T cells in tumor-associated dendritic cells (TADC). PE-treated TADCs also elicited stronger antigen-specific CTL responses. Furthermore, PE enhanced the proliferative capacity of anti-tumor T cells (increased expression of Ki67, CD137, GZMB or IFN-γ, TNF-a) and reversed T cell exhaustion (impaired CD95 or PD-1 expression). SOCS1 and SOCS3 were important negative regulators of tumor-associated immune cells during tumor development [151], PE inhibited the activation of JAK2 in SOCS1 [152].

#### Astragalus membranaceus

T cells from patients with renal cell carcinoma (RCC) exhibited multiple features of injury and depletion, as shown by the down-regulation of IL-21 expression associated with the dysfunction of CXCR5^+^ Tfh-like cells. *Astragalus membranaceus* could significantly increase the expression of IL-21 in a dose-dependent manner depending on the presence of APCs. APCs induced by astragalus extract also promoted the expression of IL-21 in Tfh-like cells. Interestingly, astragalus stimulated Tfh-like cells exhibited enhanced helper function, higher humoral responses and better CD8 T cells survival, which depended on the presence of IL-21 [153].

#### *Lycium barbarum* polysaccharide

Studies showed that *Lycium barbarum* polysaccharide (LBP), one of the active ingredients of *Lycium barbarum*, inhibited the tumor growth of H22 tumor-bearing mice. In addition, LBP could maintain high levels of T cells in peripheral blood, tumor-draining lymph nodes (TDLN), and tumor tissues. It also inhibited the increase of Tregs and TGF-β and IL-10, while promoted CD8^+^ T cells infiltration in tumor tissues, reducing the exhaustion phenotype of T cells and maintaining lymphocyte cytotoxicity [154].

#### Luteolin and apigenin

In KRAS-mutant NSCLC cancer cells, luteolin and apigenin inhibited IFN-γ-induced PD-L1 expression upregulation and STAT3 phosphorylation. PD-1 antibody combined with luteolin or apigenin treatment significantly increased the frequency and number of CD8^+^ T lymphocytes, and CD8^+^ T cells produced more IFN-γ, TNF-α and Granzyme B. Moreover, the ratio of CD8/ CD4 T cells in serum was increased, and the proportion of Treg cells (CD4^+^FoxP3^+^T cells) in spleen and blood was decreased in apigenin treatment group [155].

#### Bisdemethoxycurcumin

Bisdemethoxycurcumin (BDMC) combined with anti-PD-L1 antibody significantly prolonged the survival of bladder cancer mice, increased CD8^+^ T cells number, and promoted the secretion of IFN-γ, granzyme B, and perforin, improving the function of CD8^+^T cells. In addition, the combination group reduced the proportion of exhausted T cells and increased the number of intra-tumoral cytotoxic T lymphocyte (CTL), protecting effector T cells from exhaustion. MDSCs had a highly immunosuppressive effect in TME. The combination treatment also significantly reduced the number of MDSCs in the tumor [156].

In conclusion, according to current studies, in infection and cancer, TCM mainly reduces the expression of inhibitory receptors, inhibitory cells and cytokines, improving T cell exhaustion. However, the researches on transcription, epigenetic and metabolism related to T cell exhaustion have not been in-depth enough, which will be the focus of TCM research on improving T cell exhaustion in the future.

## Conclusions and perspective

Although T-cell exhaustion differs between infection and cancer, both share similar features, including impaired cytokine production, cytotoxicity, and elevated levels of multiple inhibitory receptors [13]. Moreover, different diseases do not exist in isolation, they are interrelated, and the occurrence of viruses will lead to the occurrence of cancer. Oncogenic viruses (oncoviruses) are implicated in approximately 12% of all human cancers [157]. For example, HBV and HCV remain the most important risk factors for HCC, and effective treatment of chronic HBV and HCV viral infections helps to reduce the incidence of viral-associated HCC [158]. T cell exhaustion is not the result of a single factor, but is affected by multiple factors. It is the result of the superposition of multiple states. Moreover, a variety of inhibitory receptors such as PD-1, PD-L1, LAG-3, TIM-3 in T cell exhaustion also affect T cell metabolism [159]. Therapeutic blockade of PD-1 reactivates the exhausted T-cell immune response by reprogramming metabolic processes, promoting proliferation, and upregulating the expression of effector molecules [5]. Studies showed that inhibition of PD-1 and PD-L1 increased glucose utilization in TME and enhanced the glycolytic activity of T cells [160]. Studies also found that after T cell exhaustion, the application of anti-PD-L1 or anti-PD-1 improved the proliferation of CD8^+^T cells, increased the production of cytokines and enhanced the cytolytic activity, reducing the viral load [32]. In addition, anti-PD-1 antibody enhanced the glycolytic capacity and effector function of exhausted CD8^+^T cells. Therapeutic blockade of anti-PD-L1 reactivated and improved the metabolic function of partially exhausted CD8^+^T cells in tumors [161]. Furthermore, TIM-3 changed T cells metabolism by disrupting the PI3K/AKT/mTOR signaling pathway [162]. The basal apoptosis rate and aerobic glycolysis of CD4^+^ T cells lacking LAG-3 were significantly increased, indicating that LAG-3 reduced the metabolic fitness of T cells [163]. All these studies suggested that the various factors of T cell exhaustion influenced each other. In addition, although exhausted T cells exhibited immune dysfunction and lose effective control of chronic infections and tumor cells, T cell exhaustion was not an irreversible terminal state [20]. Studies showed that blocking inhibitory receptors improved the functional status of T cells to a certain extent, and improved the clearance rate of viruses, tumor cells and bacteria. Blockade of PD-1, LAG-3, TIM-3, BTLA, and CTLA-4 alone or in combination with other therapies reversed T cell exhaustion during chronic infection or tumor [53, 164]. Compared with single blockade, combined blockade showed greater advantages, but the adverse reactions of combined blockade were more obvious than single blockade. In addition, it was only effective in some people, and most patients had to discontinue treatment because of serious immune-related adverse events, such as immune-related toxicity and excessive disease progression [23, 24].

TCM has a history of thousands of years in China, which is not only well respected but also has a good track record [165]. Although in recent years, TCM has been criticized for being “unscientific” or “imprecise” in oncology, an increasing number of scientific reports have confirmed the scientific basis of TCM [26]. In addition, in the study of T cell exhaustion, it was found that both TCM compounds and TCM ingredients played a role in improving T cell exhaustion through multiple channels, such as Dahuang Fuzi Baijiang Decoction [131], Yangyin Fuzheng Jiedu Prescription [132], Yangyin Fuzheng Decoction [134, 135] and HSF [147] all played a therapeutic role by inhibiting two or more IRs. Qiyusanlong Decoction [138], CKI [139], PE [151, 152], LBP [154], luteolin and apigenin [155] not only inhibited the expression of IRs, but also regulated immunosuppressive cells and immunosuppressive cytokines, so as to improve the function T cell exhaustion. In addition, there were abundant clinical research data of TCM. Shenqi Fuzheng injection had good curative effect on Chinese patients with relapsed metastatic or advanced (stage IIIB/IV) NSCLC, and significantly improved Qi deficiency constitution [166]. A meta-analysis of CKI confirmed that CKI plus transcatheter arterial chemoembolization (TACE) was superior to TACE alone in patients with unresectable hepatocellular carcinoma (UHCC) [167]. Yangyin Fuzheng Jiedu Prescription has been used in the treatment of liver cancer in the Center of Integrated Chinese and Western Medicine of Beijing Ditan Hospital (affiliated to Capital Medical University) for many years. Based on the research of the prescription has been registered in the clinical trial system (https://www.clinicaltrials.gov/), the registration number for NCT02927626. A recent meta-analysis of epidemiological studies found that apigenin intake reduced the risk of colorectal cancer [0.82(0.70–0.97)] but had no effect on colon cancer [0.88(0.69–1.13)] [168]. However, there are few TCM clinical studies on T cell exhaustion, most of which are still in the preclinical stage. However, preclinical studies of TCM show the advantages of studying T-cell exhaustion.

## Data Availability

The datasets used and/or analyzed during the current study are available from the corresponding author on reasonable request.

## Abbreviations

T cells: T lymphocytes
IFN⁃γ: Interferon⁃γ
TNF: Tumor necrosis factor
IL-2: Interleukin-2
COVID-19: Corona Virus Disease 2019
PD-1: Programmed cell death protein 1
LAG-3: Lymphocyte activation gene 3 protein
VISTA: V-domain Ig suppressor of T-cell activation
TIM-3: T cell immunoglobulin domain and mucin domain protein 3
CTLA-4: Cytotoxic T-lymphocyte-associated protein 4
COTL: Coactosin-like protein
HMGB1: High mobility group protein B1
HSPD1: Heat shock protein family member D1
HSP90AA1: Heat shock protein 90 alpha family class A member 1
BIRC5: Baculoviral IAP repeat-containing protein 5
HBV: Hepatitis B virus
HCV: Hepatitis C virus
HIV: Human immunodeficiency virus
MTB: Mycobacterium tuberculosis
PD-L1: Programmed cell death 1 ligand 1
TIL: Tumor infiltrating lymphocyte
Tex: Exhausted CD8^+^ T cells
ICIs: Immune checkpoint inhibitors
TCM: Traditional Chinese medicine
NK: Natural killer cell
DC: Dendritic cell
ZAP70: Zeta chain associated protein kinase 70
LCMV: Lymphocytic choriomeningitis virus
PI3K: Phosphatidylinositol-3-kinase
mTOR: Mammalian target of rapamycin
VSIG-3: V-Set and immunoglobulin domain containing 3
APC: Antigen-presenting cell
Gal-9: Galectin-9
CEACAM1: Carcinoembryonic antigen-related cell adhesion molecule 1
HMGB-1: High-mobility group box
PtdSer: Phosphatidylserine
MHC II: Major histocompatibility complex class II
FGL-1: Fibrinogen-like protein 1
Gal-3: Galectin-3
DNMT3A: DNA methyltransferase 3 alpha
TOX: Thymocyte selection-associated high mobility group box
Eomes: Eomesodermin
CAR-T: Chimeric antigen receptor T-cell immunotherapy
LDHA: Lactate dehydrogenase A
H3K9: H3 lysine 9 residue
IDO: Indoleamine-pyrrole 2,3-dioxygenase
MDSCs: Myeloid-derived suppressor cells
TAMs: Tumor-associated macrophages
mtROS: Mitochondrial reactive oxygen species
PGC-1α: Peroxisome proliferator-activated receptor-γ coactivator 1α
Blimp-1: Blymphocyte-induced maturation protein 1
HIF: Hypoxia-inducible factor
Tregs: Regulatory T cells
PPD: Purified protein derivative
YFJP: Yangyin Fuzheng Jiedu Prescription
DFB: Dahuang Fuzi Baijiang Decoction
CCR2: Chemokine C–C-motif receptor 2
QYSL: Qiyusanlong Decoction
SFI: ShenQi FuZheng Injection
*A. indica*: *Azadirachta* *indica*
SEB: Staphylococcal Enterotoxin B
GK: Guttiferone K
Tc: Cytotoxic T cells
Th: Helper T cells
HSF: Hirsutella sinensis fungus
APS: Astragalus membranaceus polysaccharide
RCC: Renal cell carcinoma
PE: Pinellia pedatisecta Schott extract
LBP: Lycium barbarum polysaccharide
BDMC: Bisdemethoxycurcumin
CTL: Cytotoxic T lymphocyte
TCRs: T cell receptors
